# Effects of different anesthetic regimens on postoperative cognitive function of elderly patients undergoing thoracic surgery: a double-blinded randomized controlled trial

**DOI:** 10.1186/s13019-024-02939-w

**Published:** 2024-06-27

**Authors:** Li Xie, Xin Wei, Keqiang He, Sheng Wang, Min Xu

**Affiliations:** Department of Anesthesiology, Division of Life Sciences and Medicine, The First Affiliated Hospital of USTC, University of Science and Technology of China, Hefei, 230001 Anhui China

**Keywords:** Dexmedetomidine, Postoperative cognitive dysfunction, Propofol, Sevoflurane, Thoracic surgical procedure, Cognitive dysfunction, Postoperative cognitive complications, Neuropsychological tests

## Abstract

**Objective:**

Postoperative cognitive dysfunction (POCD) is a serious surgical complication. We assessed the different POCD incidences between anesthesia using sevoflurane and sevoflurane combined with dexmedetomidine, with propofol-based sedation in elderly patients who underwent a thoracic surgical procedure.

**Methods:**

A total of 90 patients aged 65 to 80 years old who underwent a thoracic surgical procedure at our hospital and 15 nonsurgical participants as controls, were enrolled in this study. Patients were divided in a randomized 1:1:1 ratio into 3 groups. All participants were randomized into a trial with three anesthesia groups (P, PS, PSD) or a control group (C) of healthy matches. All trial groups received distinct anesthetic combinations during surgery, while controls mirrored patient criteria.Group P (propofol and remifentanil were maintained during the surgery), Group PS (propofol, remifentanil, and sevoflurane were maintained during the surgery), and Group PSD (propofol, remifentanil, sevoflurane, and dexmedetomidine were maintained during the surgery).All participants were rated using a series of cognitive assessment scales before and three days after surgery. All participants were interviewed over the telephone, 7 days, 30 days, and 90 days postoperatively.

**Results:**

POCD incidences in the PSD (combined anesthetization with propofol, sevoflurane, and dexmedetomidine) group was significantly lower than that in the PS (combined anesthetization with propofol and sevoflurane) group, 1 day post-surgery (10.0% vs. 40.0%, *P* = 0.008), and the results were consistent at 3 days post-surgery. When the patients were assessed 7 days, 30 days, and 90 days postoperatively, there was no significant difference in POCD incidence among the three groups. Multivariate logistic regression analysis of POCD one day after surgery showed that education level was negatively correlated with incidence of POCD (*P* = 0.018) and single lung ventilation time was positively correlated with incidence of POCD (*P* = 0.001).

**Conclusion:**

For elderly patients who underwent a thoracic surgical procedure, dexmedetomidine sedation shows an obvious advantage on improving short-term POCD incidence, which is caused by sevoflurane.

## Introduction

Cognitive disorders after anesthesia and surgery are associated with morbidity and increasing mortality. Such postoperative cognitive dysfunction (POCD), with wide diagnostic criteria, tends to occur in elderly patients who have received general anesthesia. The presence of POCD within weeks or months after surgery is associated with undesirable outcomes, such as increasing demand for social support, poor quality of life, and mortality [1–4]. 

The etiology and pathogenesis of the increase in POCD is of concern and involves several affecting factors—general anesthesia is now considered and has been accepted as an independent risk factor. Sevoflurane, an inhalable anesthetic is a common maintenance narcotic drug for clinical general anesthesia, and its potential risk of POCD in elderly patients is widely recognized [5]. The neurotoxicity of inhaled anesthetics, including amyloid deposition in nerve cells, has proven to cause cognitive dysfunction [6, 7]. Propofol, another general anesthetic, has attracted extensive controversy on whether it can promote nerve cell apoptosis, especially at different stages of the life cycle and in certain morbid states. The neuroprotection, neurotoxicity, or indifferent effects, depend on the selection of anesthetics and scheme of anesthesia [8]. A prospective study suggested that sevoflurane was relevant to POCD incidence in elderly patients [9]. However, in another randomized controlled trial, the incidence of early postoperative POCD in elderly patients receiving propofol anesthesia was lower than that of sevoflurane [10]. 

Although the mechanisms underlying POCD have not been fully elucidated, researchers have been striving to reduce its incidence. A recent study by Glumac and colleagues suggests that inflammation and stress responses may be involved in the development of POCD. The preemptive use of dexamethasone during surgery may potentially improve the occurrence of POCD [11]. Another promising focus for improving POCD is the dexmedetomidine, a α2-adrenergic agonist, has a range of positive effects in the perioperative period, such as residual opioid and reduction in the narcotics dose, and even induces positive neuroprotective effects, which has been confirmed with animal experiments [12–15]. A similar scenario shows that dexmedetomidine reduces the incidence of delirium better than propofol and midazolam, which caused alleviation of systemic stress response in intensive care unit (ICU) patients [16]. The phenomenon of lower POCD incidence based on intraoperative dexmedetomidine has risen to prominence in recent years.

Studies have shown that POCD incidence in elderly patients undergoing thoracic surgery ranged from 32.0 to 49.4%. The possible reasons were that ventilator-induced lung injury and systemic inflammatory response caused by ventilation during surgery, as well as unbalanced ventilation perfusion ratio, directly decreased cerebral oxygen saturation and caused cognitive-related area injury [17, 18]. 

The primary objective of the current study is to investigate the different occurrence of POCD in elderly patients who received general anesthesia based on sevoflurane or propofol, while undergoing thoracic surgery. Furthermore, we hope to demonstrate the hypothesis that anesthesia management in the ICU assisted by dexmedetomidine shows a lower incidence of POCD in elderly patients than that without it. Endpoints consist of cognitive function before operation and at 1 day, 3 days, 7 days, 30 days, and 90 days after the surgery.

We hypothesized that the use of dexmedetomidine in elderly thoracic surgery patients leads to significantly reduced postoperative cognitive dysfunction compared, caused by sevoflurane, as mainly measured by MMSE, IQCODE.

## Patients and methods

### Study design

This study was conducted from July 2021 to Juliy 2022, and it was a prospective, randomized, double-blind controlled trial, conducted in one center, and was approved by the ethics committee of our hospital (No.2021-ky112) and registered with the Chinese Clinical Trial Registry (NO.ChiCTR2200058066) on March 28, 2022 (which was a retrospective registration). Patients consented to their interviews with the leading anesthesiologists the day before surgery and we obtained informed consent from patients or their legally recognized relatives.

### Group design

All eligible participants were divided into the trial group and control group. The patients who underwent surgery were enrolled in the trial group, and randomly divided into three groups, delineated as Group P (propofol and remifentanil were maintained during the surgery), Group PS (propofol, remifentanil, and sevoflurane were maintained during the surgery), and Group PSD (propofol, remifentanil, sevoflurane, and dexmedetomidine were maintained during the surgery). All patients in the trial group were administered anesthesia following the same induction regimen.

Control subjects in the Control Group (Group C) had congruously inclusive and exclusive criteria with eligible patients, and were recruited from amongst their healthy relatives or friends.

### Study participants

Potential patients were screened at the same time as their preoperative visits. Eligible participants were expected to stay in the hospital for at least three days after surgery and had to agree to postoperative telephone interviews at home. All recruited patients who suffered from lung disease and were undergoing major elective thoracic surgery including video-assisted thoracoscopic lobectomy, wedge resection of the lung, and segmentectomy, were given general anesthesia. Inclusion criteria were: (1) aged 65 to 80 years; (2) primary pulmonary diseases without chemoradiotherapy; (3) scheduled to undergo surgery under general anesthesia; (4) American Society of Anesthesiologists (ASA) grade I, II, and III; (5) normal linguistic competence in Chinese (especially in speaking and listening); and (6) able to complete cognitive function tests independently. Exclusion criteria were:1) unwillingness to enroll in the study; 2) Cognitive impairment was detected preoperatively; 3) surgical history of cardiac surgery or neurosurgery; 4) severe mental disorder as well as hearing and visual disabilities; 5) abuse or dependence on alcohol or psychotropic drugs (tranquillizers or antidepressants); 6) severe hepatorenal dysfunction (Child-Pugh stage C or maintenance of renal replacement therapy); (7) cachexia or systemic malignant diseases; or (8) aborted operation.

### Randomization and blinding

Eligible patients undergoing thoracic surgery were randomly and equally allocated to three groups—(Group P)—propofol-dependent general anesthesia, (Group PS)—sevoflurane inhalational-based general anesthesia combined with propofol intravenous anesthesia, and (Group PSD)—general anesthesia administered in combination with propofol, sevoflurane, and dexmedetomidine. This was done using the sealed envelope method. Numbers of the group allocation were hidden from participants, investigators, and anesthetists in enclosed envelopes and were revealed as the patients were wheeled into the operating room.

### Perioperative interview and cognitive examination

Demographic data (sex, age, BMI, and education level) and clinical characteristics (ASA, surgical diagnosis, and medical history) of patients was collected the day before surgery. Preoperative assessment of cognitive dysfunction through patient visits and administration of the MMSE test. Neurocognitive data were recorded using Mini-Mental State Examination (MMSE), Informant Questionnaire on Cognitive Decline in the Elderly (IQCODE), Digit Span Test (DST), Trail Making Test (TMT), and Verbal Fluency Test (VFT).

### Anesthesia procedure

Three experienced anesthesiologists handled all patients, who were intubated following induction of anesthesia with a double lumen tube, which was located by fibrous bronchoscope. Patients in Group P received targeted and controlled infusion of propofol (2 ~ 3 ug/ml) for anesthesia maintenance; patients in Group PS received targeted and controlled infusion of propofol (2 ~ 3 ug/ml) and sevoflurane (0.5 MAC) for anesthesia maintenance; and patients in Group PSD received targeted and controlled infusion of propofol (2 ~ 3 ug/ml), sevoflurane (0.5 MAC), and dexmedetomidine (Loading dose 1 ug/kg (15 min) maintenance dose 0.3 ug/kg/h) for anesthesia maintenance [19]. And these three drugs were pumped simultaneously. All three surgical groups were targeted with remifentanil (2 ~ 4 ng/ml), and cisatracurium was injected intermittently.

We maintained the bispectral index (BIS VISTA™, 185-0151-USA, 15 Hampshire Street, Mansfield, MA, USA) between 40 and 60, the mean arterial pressure (MAP) within 20% of baseline (the day of registering into the hospital), and the heart rate (HR) in the range of 50 to 100 bpm. We maintained stable hemodynamic parameters by giving noradrenaline to increase MAP and nitroglycerin to decrease it, and increased HR by using atropine or decreased it by using esmolol. Hemodynamic parameters were recorded every 5 min during the entire anesthesia procedure. Patients recovered in the post-anesthesia care unit (PACU) after the surgery was completed. Postoperative analgesia was performed by patient-controlled intravenous analgesia (PICA) (100 ml of normal saline containing 2 mg/kg flurbiprofen axetil and 2 µg/kg sufentanil, with a first dose of 0.04 mg/kg flurbiprofen axetil and 0.04 ug/kg sufentanil, and a maintenance dose of 0.05 mg/kg/h flurbiprofen axetil and 0.05 ug/kg/h sufentanil, for the last 2 days).

### Evaluation of postoperative cognitive function

Evaluation tests such as MMSE, DST, TMT-A, and VFT were used to assess the cognitive function at the endpoints of 1 day and 3 days after treatment. MMSE is a 30-point questionnaire, which includes simple tasks in time and place, testing mathematics, linguistic application and comprehension, and so on. Higher scores suggest better cognitive function [20]. DST is a subtest of the Wechsler Adult Intelligence. A higher score indicates better attention and short-term memory of participants [21]. TMT is administrated to measure visuospatial and executive ability, and used to screen cognitive impairment [22, 23]. Lastly, VFT is mainly applied to assess linguistic, semantic memory, and executive ability of participants, including semantic, speech, and movement fluency, particularly semantic fluency extensively used in China [24–26]. 

The Informant Questionnaire on Cognitive Decline in the Elderly (IQCODE) was recommended to assess cognitive function at the endpoints of 1 day, 3 days, 7 days, 30 days, and 90 days after surgery as a supplement to MMSE. Moreover, IQCODE has a key advantage over MMSE, in that it is rarely influenced by the target’s intelligence or education history, making it a important questionnaire to evaluate cognitive dysfunction [27–29]. 

### Sample size calculation

**Fig. 1:**
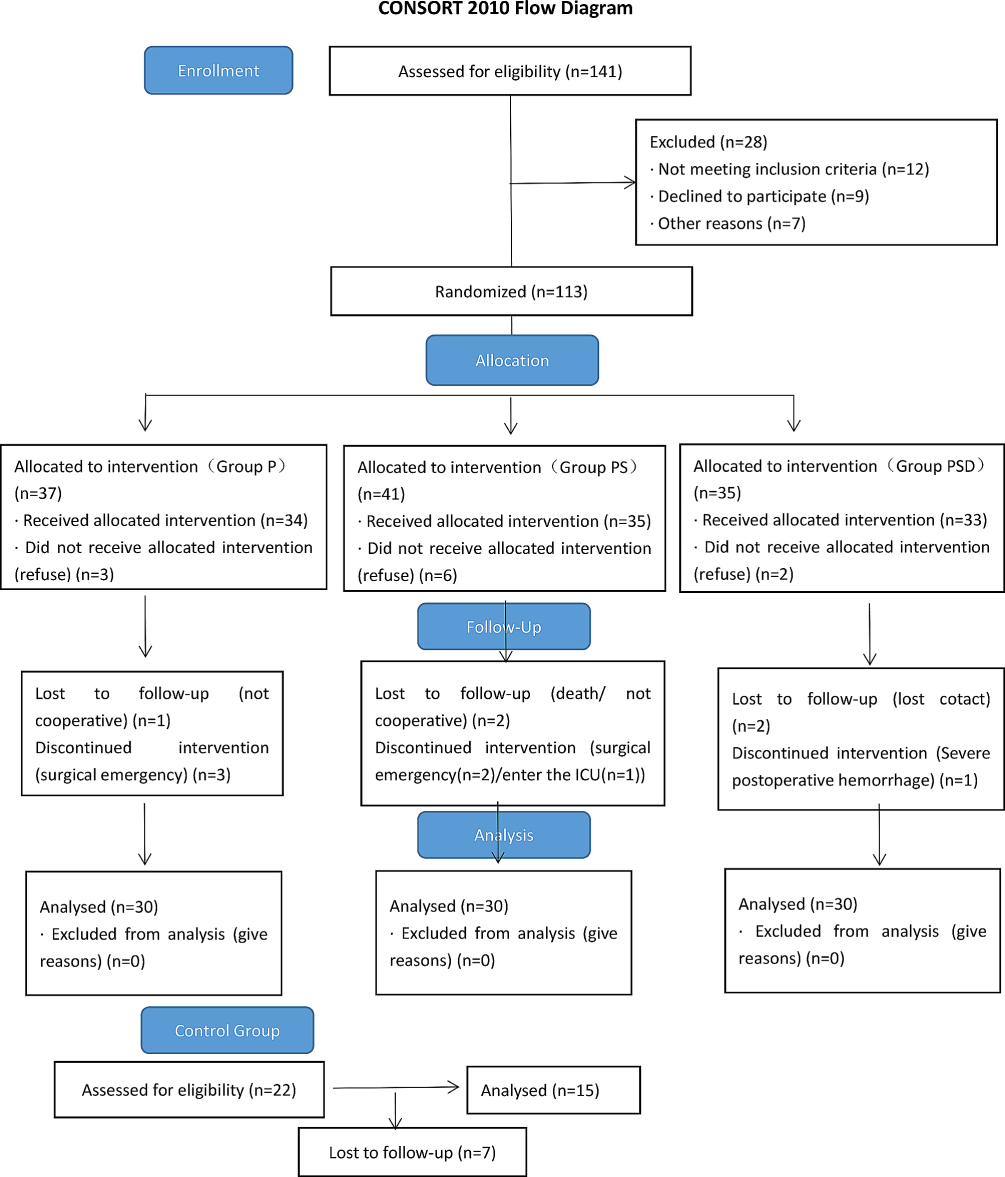
The flow chart of the study

According to previous studies, the incidence of early POCD (1 day after surgery) was about 49.4% [17]. We hypothesized that the incidence of POCD is reduced by 30% in the Group PSD with an a-error and power set at 0.05 (2 side) and 80%, respectively. The sample size involved in the study had at least 87 patients. A total of 90 patients were recruited in this study and randomly assigned to three groups in the ratio of 1:1:1. The ratio of surgical patients registered to healthy volunteers in Group C was 6:1. Figure 1 is the flow chart of this study.

### Outcomes and statistical analyses

The primary outcome of this study was the incidence of POCD at 1 day following surgery, while incidences were also recorded at 3, 7, 30, and 90 days after surgery. The test results were adjusted by an established formula, which was used to assess POCD. The formula was used to create a Z score to indicate cognitive status of participants. A patient was classified as exhibiting cognitive impairment or POCD when the Z score was ≥ 1.96 (*Z*=(*X*-*X*_reference_)/SD_control_, *X* is the difference between postoperative and basic tests score; *X*_reference_ is the difference between the tests scores of patients in Group C and baseline at the corresponding time point; and SD_control_ is the standard deviations (SD) of those changes in Group C) [30, 31]. 

All statistical analyses were conducted using SPSS version 24.0 for Windows (SPSS Inc., Chicago, IL). The unpaired T-test was used to analyze continuous variables when they conformed to normal distributions, and the Mann-Whitney U test or Kruskal-Wallis H test was used in continuous variables with abnormal distributions. The Fisher’s exact, chi-square tests, or correction were used to compare proportions, and multinomial logistics regression analysis was used to calculate the odds ratios (ORs). The *P* values were 2-sided, and *P* values that were less than 0.05 were considered significant.

## Results

### Participant characteristics

From July 2021 to November 2021, 107 patients were screened for eligibility, and of these, 90 completed this clinical trial. Finally, 30 patients received propofol-based anesthesia (Group P), 30 patients received anesthesia based on propofol and sevoflurane (Group PS), and 30 patients received anesthesia based on propofol, sevoflurane, and dexmedetomidine (Group PSD). At 90 days after the surgery, no patient died or were lost to follow-up.

The demographic and clinical preoperative characteristics of the surgical patients in Group P, Group PS, Group PSD, and Group C showed no significant differences. There were no significant differences among surgical patients in Group P, Group PS, and Group PSD in clinical characteristics throughout the anesthesia period (Table 1). There were no significant differences in perioperative hemodynamic parameters among surgical patients in Group P, Group PS, and Group PSD (Table 2).

**Table 1:** Demographic and clinical characteristics of the participants

	Group P(*n* = 30)	Group PS(*n* = 30)	Group PSD(*n* = 30)	Group C(*n* = 15)	*P* value
Age(years)	70.80 ± 4.5	70.47 ± 4.44	68.53 ± 3.03	70.53 ± 3.27	0.117
Gender(male)	12(40.0%)	18(60.0%)	14(46.7%)	9(60.0%)	0.373
BMI(kg/m^2^)	23.32 ± 3.18	23.78 ± 2.84	23.49 ± 3.62	23.47 ± 2.72	0.953
Education(years)	5.93 ± 2.38	5.40 ± 2.43	5.47 ± 1.94	5.13 ± 2.20	0.660
ASA status					0.747
I	5(16.7%)	5(16.7%)	4(13.3%)	4(26.7%)	
II	20(66.7%)	20(66.7%)	20(66.7%)	9(60.0%)	
III	5(16.7%)	5(16.7%)	6(20.0%)	2(13.3%)	
NYHA classification					0.695
I	17(56.7%)	19(63.3%)	20(66.7%)	-	
II	9(30.0%)	9(30.0%)	7(23.3%)	-	
III	4(13.3%)	2(6.7%)	3(10.0%)	-	
MMSE(score)	23.70 + 3.86	23.73 + 3.52	23.47 + 3.71	25.93 + 2.19	0.142
Hypertension	15(50.0%)	12(40.0%)	12(40.0%)	4(13.3%)	0.512
Diabetes	4(13.3%)	6(20.0%)	3(10.0%)	2(13.3%)	0.729
Coronary heart disease	4(13.3%)	3(10.0%)	4(13.3%)	2(6.7%)	0.935
Surgery history	12(40.0%)	12(40.0%)	18(60.0%)	5(16.7%)	0.626
Postoperative complications					
Cough	30(26.7%)	30(36.7%)	30(30.0%)	-	0.696
Pain	14(46.7%)	13(43.3%)	16(53.35)	-	0.732

Data are presented as the number(%) or mean ± SD. BMI: body mass index; NYHA: New York Heart Association; MMSE: Mini-Mental State Examination

**Table 2:** Characteristics of patients in preoperative period

	Group *P*	Group PS	Group PSD	*P* value
Duration of anesthesia (min)	181.76 + 94.89	142.20 + 43.17	145.83 + 66.08	0.277
Duration of surgery(min)	165.80 + 96.96	113.73 + 40.68	115.36 + 64.80	0.058
Intraoperative infusion in surgery(ml)	1280.00 + 965.04	1043.33 + 315.88	931.67 + 346.53	0.446
Estimated blood loss(ml)	53.50 + 55.86	29.33 + 21.12	42.33 + 56.67	0.488
Urine volume(ml)	308.00 + 328.85	219.66 + 211.62	202.33 + 206.43	0.225
MAP(mm Hg)				
Entering room	97.81 + 10.69	94.92 + 10.07	100.42 + 12.17	0.163
Inbutation	80.02 + 9.69	86.72 + 10.48	85.56 + 11.92	0.811
Skin incision	83.58 + 11.25	83.33 + 12.45	87.94 + 9.91	0.150
End of surgery	84.42 + 8.84	85.44 + 9.81	87.41 + 7.47	0.536
Extubation	97.13 + 13.65	97.00 + 16.69	96.29 + 9.05	0.822
Mean MAP during surgery period	81.63 + 9.55	77.44 + 9.24	81.08 + 9.61	0.149

#### Incidence of POCD after surgery

(Table 3).

**Table 3:** Incidence of POCD Group P, Group PS, Group PSD

	Group P(*n* = 30)	Group PS(*n* = 30)	Group PSD(*n* = 30)	*P* value (*P* vs. PS)	*P* value (*P* vs. PSD)	*P* value (PS vs. PSD
POCD at 1 day	4(13.3%)	12(40.0%)	3(10.0%)	0.021	0.690	0.008
POCD at 3 days	6(20.0%)	11(36.7%)	3(10.0%)	0.155	0.682	0.015
POCD at 7 days	1(3.3%)	3(10.0%)	2(6.7%)	0.305	0.557	0.643
POCD at 30 days	0	1(3.3%)	1(3.3%)	0.317	0.317	-
POCD at 3 months	0	0	0	-	-	-

### Incidence of POCD on day 1 post-surgery

On day 1 post-surgery, 21.1% of patients who had undergone surgery had severe POCD. Patients in Group PS had a higher incidence of POCD than patients in the Group PSD at the corresponding time point (40.0% vs. 10.0%, *P* = 0.008); the different incidences of POCD between Group P and Group PSD showed no significant difference (13.3% vs. 10.0%; *P* = 0.690).

### Evaluation of POCD at 3 days post-surgery

At 3 days post-surgery, 22.2% (20 out of 90 patients who received surgery) were considered to suffer from POCD, and there was no significant difference with occurrence in cognition impairment at day 1 post-surgery (22.2% vs. 21.1%, *P* = 0.857). Patients in Group PS showed worse performance in terms of POCD than patients in Group PSD at the corresponding time point, with 36.7% (11 out of 30 patients) versus 10.0% (3 out of 30 patients) (*P* = 0.015); and had no different incidences than at day 1 post-surgery (36.7% vs. 40.0%, *P* = 0.792). While patients who received surgery in Group PSD had no different incidences of POCD than Group P at 3 days post-surgery (10.0% vs. 20.0%, *P* = 0.682).

### Evaluation of POCD at 7 days post-surgery

At 7 days post-surgery, only 6 out of 90 patients (6.7%) who underwent surgery suffered from POCD, and the healthy controls in this study had no occurrence of cognition impairment at the corresponding time point. The different incidences of POCD among the three groups showed no significant difference (*P* = 0.589). The incidence of POCD at 7 days post-surgery showed a significantly lower rate in Group PS, with 10.0% than 36.7% at 3 days after surgery (*P* = 0.015).

### Evaluation of POCD at 30 days and 90 days post-surgery

Two patients were in a persistent state of POCD 30 days post-surgery. One patient died from severe pulmonary complications at 63 days after surgery. There was no occurrence of cognition impairment at 90 days post-surgery.

### Multivariate logistic regression analysis of POCD

**Fig. 2:**
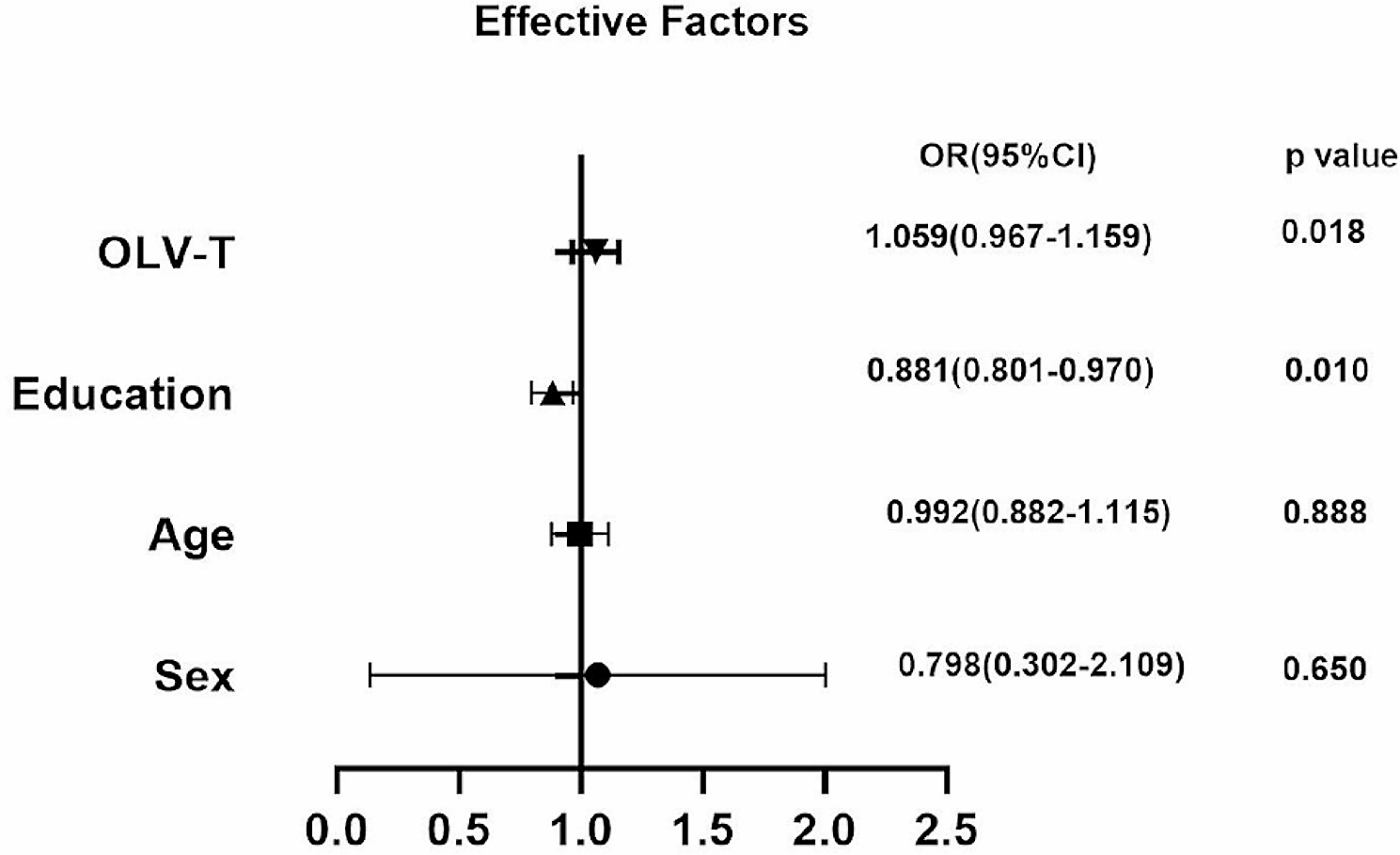
Multivariate logistic regression analysis of POCD at 1 day after surgery

Factors such as age, sex, education level, and single lung ventilation time (OLV-T) were included in the multi-factor logistic regression equation. The results showed that the higher the education level, the lower the risk of POCD at one day after surgery, with statistical significance (OR = 0.405, 95% CI 0.192–0.858, *P* = 0.018); the longer the duration of single lung ventilation, the higher the risk of POCD at one day after surgery (OR = 1.059, 95% CI 0.967–1.159, *P* = 0.001). Age, sex, education level, and single lung ventilation duration had no statistical significance on POCD on the third day, the seventh day, 30 days, and 90 days after surgery (*P* > 0.05).(Fig. 2).

## Discussion

The results of our study revealed that elderly patients undergoing thoracic surgery had an obviously high occurrence of POCD—sevoflurane contributed to the development of POCD, while dexmedetomidine offered the benefit of ameliorating it. As time went on, the incidence of POCD ameliorated consistently and returned to preoperative levels at seven days post-surgery.

POCD is a disorder of the mind, which is a reversible and fluctuating acute mental disorder syndrome that occurs within a few days after surgical anesthesia [32]. Thoracic surgery has the traits of severe trauma, stress reaction, postoperative pain, and grievous systemic inflammatory response, and patients undergoing surgery tend to suffer from a range of perioperative complications. This is particularly evident in elderly patients owing to their slow and unsteady gait and decline in physiological function [33, 34]. POCD is prone to occur in elderly patients undergoing thoracic surgery, with a published prevalence of 10.6–36.54%. Our study results demonstrated that POCD at one day post-surgery developed in 13.3% of patients in Group P; this was in concordance with published incidence (18.2%) in other studies, where patients received propofol anesthesia [8, 35]. Incidence of POCD in Group PS was 40.0%, which is analogous with previous trials where patients underwent sevoflurane anesthesia [36]. The potential manifestation of the higher incidence of POCD in Group PS are not clear, which may be due to the changing of integrity of the blood brain barrier (BBB). Animal studies showed that the BBB integrity is associated with biological aging and there is an age-related reduction in tight junction proteins that causes increasing permeability [37, 38]. Synchronously, high concentration of sevoflurane further causes more serious intracerebral oxidative stress response. Subsequent results indicated that POCD in patients in Group P had no significant difference with those in Group PS at 7 days, 30 days, and 90 days post-surgery.

Cerebral perfusion, on account of physiological senility or anesthesia, is an important risk factor for POCD [39], however, a previous clinical study indicated that intraoperative hypotension of the cerebrum was not correlated with POCD in elderly patients who were operated under general anesthesia [40]. Cardiac function is a crucial factor for maintaining intraoperative hemodynamic stability and cerebral perfusion. However, a recent clinical survey revealed that sevoflurane-based anesthesia conserved left ventricular function in coronary heart disease patients who suffered from severe illness [41]. Furthermore, intraoperative hemodynamic parameters of patients were tightly regulated within the target scope during the entire period when they were under anesthesia, maintaining proper depth of sedation by monitoring bispectral index (BIS) values, and modulating vasoactive agents to ensure abundant perfusion of vital organs. Perioperative hemodynamic parameters showed no significant differences between sevoflurane-based and propofol-based anesthesia. In the present study, findings indicated that intraoperative hemodynamic parameters did not have significant difference in patients between Group P and Group PS. We found that intraoperative cerebral hypoperfusion is not associated with POCD in elderly patients undergoing thoracic surgery in this research.

Although previous clinical studies showed that general anesthesia had a positive impact on POCD in elderly patients undergoing non-cardiac surgery, the research was limited when it came to observing thoracic surgery [8, 42]. Thoracic surgery is frequently associated with various perioperative complications, and it is currently confirmed to be associated with POCD, which is probably caused by severe surgical stress reactions and surgical trauma that impairs physiological capacity, leads to cytokine imbalance, and enhances the inflammatory response [43–45]. The findings of the present study showed a significantly higher incidence of POCD (40.0%) than the incidence of POCD after non-cardiothoracic surgery observed by other researchers (29.1%) [36]. We concluded that thoracic surgery was an independent risk factor in performance of POCD.

Thoracic surgery is usually accompanied with special ventilation methods, including single-lung or two-lung ventilation, which causes immediate lung injury and systemic inflammatory response and aggravates postoperative cognitive function. Additionally, an abnormal ratio of pulmonary ventilation/perfusion can directly reduce patients’ cerebral oxygen saturation and damage the cognitive region based on the hypothesis of neuroinflammation-related POCD [43]. Strategies for alleviating inflammation and oxidative stress can ameliorate cognitive function of patients undergoing thoracic surgery. Research revealed that as an α2-adrenergic agonist, dexmedetomidine had an individual sedative and anesthetic effect, did not induce respiratory depression, and provided hemodynamic stabilization and neuroprotection by moderating systemic inflammatory response during the perioperative period. Dexmedetomidine can inhibit the release of serum inflammatory factors, such as IL-6, TNF-α, AKT, and PI3K [35, 42]. Results of correlative analysis indicate that there is a negative correlation between the MMSE score and the expression levels of these specific inflammatory factors [46]. In the present study, the incidence of POCD in the Group PSD was significantly lower than Group PS (10.0% vs. 40.0%), and dexmedetomidine can improve MMSE score. We known that POCD is relevant to the expression levels of inflammatory factors [35, 42]. It is proposed that dexmedetomidine may decrease the incidence of POCD in elderly patients undergoing thoracic surgery by modulating specific inflammatory factors, but it also needs further verification in subsequent clinical trials [47]. 

In this study, we followed up the patients through telephonic interviews and conducted the TMT to evaluate the state of cognition. The results showed that patients in the three groups did not have significant differences in the incidence of POCD at 7 days, 30 days, and 90 days post-surgery.

In addition, factors such as age, gender, education level, and duration of single lung ventilation were also included in the logistic regression analysis. The results showed that the education level was a favorable factor in the prevention of POCD, while one-lung ventilation during thoracic surgery was an independent risk factor for it. The longer the duration of single lung ventilation, the higher the risk of POCD. This is similar to previous studies [48, 49]. 

There are still several limitations to this study. The relatively small sample size and a single-center clinical trial may be potential factors that lead to a biased result. Furthermore, the long-term state of cognitive function was not estimated at the 90-day follow-up after surgery, due to the difference of living environment, community environment, family and other factors may cause the change of mental state of patients. And follow-up cannot be as convenient, efficient, and comprehensive as during hospitalization. In addition, different surgeries may have different risk for POCD occurrence, but the risk differences caused by different surgical types were not taken into consideration in this study, which may cause deviation in the results. Further research will be carried out according to different types of surgery. Finally, patients encountering POCD in this study needed multidisciplinary interventions for recovery; but no relevant data was recorded and analyzed. This may lead to bias in the post-surgery follow-up.

## Conclusions

This clinical trial proved that sevoflurane-based general anesthesia led to a higher incidence of POCD in elderly patients who had undergone thoracic surgery. Dexmedetomidine reduced the incidence of POCD in elderly patients undergoing thoracic surgery. The selection of anesthesia, including sevoflurane-dependent, sevoflurane combined with propofol, and general anesthesia administered in combination with propofol, sevoflurane, and dexmedetomidine did not influence the incidence of POCD at 7 days, 30 days, and 90 days post-surgery.

## Data Availability

All data generated or analysed during this study are included in this article. Further enquiries can be directed to the corresponding author.
